# Formation of G-quadruplex structure in supercoiled DNA under molecularly crowded conditions†

**DOI:** 10.1039/c9ra06370f

**Published:** 2019-08-21

**Authors:** Dawei Li, Peiwen Peng, Zhaoqi Yang, Bei Lv

**Affiliations:** The Southern Modern Forestry Collaborative Innovation Center, College of Biology and the Environment, Nanjing Forestry University 159 Longpan Road Nanjing 210037 China dwli@njfu.edu.cn; Jiangsu Key Laboratory for Biofunctional Molecules, College of Life Science and Chemistry, Jiangsu Second Normal University Nanjing 210013 China lvbei@jssnu.edu.cn; School of Pharmaceutical Sciences, Jiangnan University Wuxi 214122 China

## Abstract

G-quadruplex is a secondary structure of nucleic acids that plays crucial roles in many significant biological processes. Potential G-quadruplex-forming sequences exist widely in various regions of the genome such as telomeres and gene promoters. In spite of the fact that G-quadruplex can be readily assembled from a single-stranded segment of DNA, its formation from duplex DNA is very difficult under physiological conditions because Watson–Crick interactions in guanine rich segments need to be weakened first. It is demonstrated in our studies that intrastrand G-quadruplex generated from a perfectly matched guanine-rich duplex in a circular DNA as a result of significant quadruplex stabilization and duplex destabilization created by the combined actions of negative DNA supercoiling and molecular crowding conditions.

## Abstract 2

It is demonstrated that G-quadruplex generated from G-rich duplex in a circular DNA as a result of quadruplex stabilization and duplex destabilization created by the combined actions of negative DNA supercoiling and molecular crowding condition.

## Graphical abstract

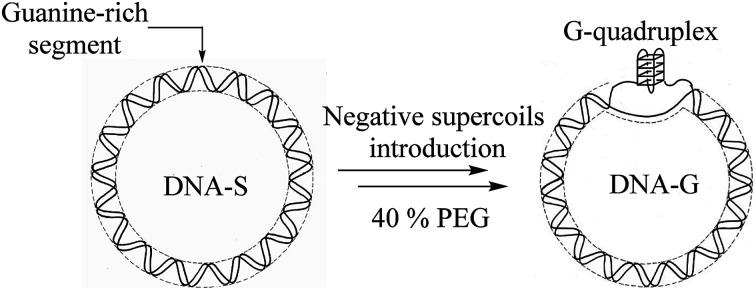

## Introduction

G-quadruplex is a nucleic acid secondary structure that is composed of a planar arrangement of four guanine residues and plays crucial roles in many significant biological processes.^1,2^ Potential G-quadruplex-forming sequences exist widely in the genomes of various organisms.^3,4^ It has been estimated that there are about a million guanine-rich sites in the genomes of eukaryotic cells that have the potential of forming G-quadruplex structures, especially in the regions of telomeres and gene promoters.^5–7^ Different from the 3′ overhang in telomeres where G-quadruplex structures can be readily assembled from single-stranded DNA, the formation of G-quadruplex in gene promoters cannot proceed in a spontaneous manner under mild conditions because it is blocked by the complementary strands of G-rich segments and the adjacent duplex regions. Therefore, an additional driving force is needed to weaken the Watson–Crick base pairing within G-rich duplex regions in order to facilitate the generation of the intrastrand G-quadruplex structures *via* a cyclic Hoogsteen hydrogen-bonding arrangement.^8–10^

DNA supercoiling, on the other hand, is a tertiary structure of nucleic acid and it means that the molecular architecture of DNA exists in space in a self-twisted fashion. DNA stored in living organism mainly exist in negatively supercoiled conformation.^11^ It is believed that the global alteration of DNA structure in supercoiling is directly caused by change of helical property of duplex DNA molecules.^12^ Watson–Crick interaction in negative supercoiling is weaken through unwinding DNA double helix to facilitate the formation of denaturation bubbles during the course of replication and transcription and it may also provide a chance to generate the intrastrand secondary structures. It has been reported that Mg^2+^-dependent supercoiling-induced structural transition takes place a in a plasmid DNA with G-rich inserts.^13^ In our previous studies, the DNA gyrase-driven formation of G-quadruplex structure in a negatively supercoiled plasmid was demonstrated.^14^ However, Sekibo and Fox reported that negative supercoiling alone is not sufficient to drive G-quadruplex formation.^15^ We speculated that those conflicting conclusions may result from the fact that purified DNA gyrase can induce a superhelical density of *σ* ≈ −0.1 in a test tube and this level of supercoiling is about twice that observed for DNA purified from cells.^16^ The higher levels of supercoils achieved by our previous *in vitro* study promoted the G-quadruplex extrusion.^14^ On the other hand, the intracellular environment is the presence of high concentrations of macromolecules (200–400 mg ml^−1^) in cells that eventually occupy up to 40% of a cell's volume.^17^ Molecular crowding condition is a reality of intracellular environment that can be created by 40% (w/v) PEG in a test tube.^18^ It has been reported that molecular crowding condition can destabilize duplex and promote and stabilize quadruplex formation.^19–21^ In the current studies, three G-rich sequence containing DNA topoisomers with “physiological” superhelical densities^11^ were engineered by PNA invasion approach.^22^ The possibility of G-quadruplex formation in those DNA topoisomers under molecular crowding condition created by PEG was investigated. The results showed that G-quadruplex only appears in the DNA with the superhelical density of −0.05 and −0.06. G-quadruplexes generated in circular DNAs were detected and analyzed directly at the single-molecule level through using atomic force microscopy and its associated software, as well as through electrophoretic analyses and enzymatic assays.

## Results and discussion

**Fig. 1:**
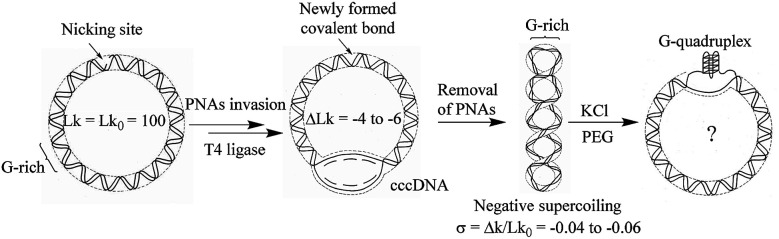
Diagrammatic illustration of G-quadruplex formation in supercoiled G-rich containing circular DNA.

In order to study the formation of G-quadruplex in circular DNA with different superhelical density, the substrate DNA circle with a G-rich sequence and several PNA binding sites was designed. The DNA circle (DNA-S, 1040 bp) has a 49 bp fragment of the murine Sγ3 switch region, in which a potential G-quadruplex forming sequence (see ESI† for sequence information) exists.^23^ The synthetic route toward circular DNA follows our previous reports^14^ and is given in Fig. S1.† Precise engineering DNA topological structures were accordingly performed using the nicking site-containing DNA through the PNA invasion strategy as shown in Fig. 1 and S2.† Three bis-PNAs^24^ (PNA 1–3) were design and synthesized to open the double helix in DNA substrate (see ESI† for detail information). There are several PNA binding sites designed in the substrate DNA-S and DNA topoisomers (*σ* = −0.04, −0.05 and −0.06) can be produced by using different combinations of PNAs.

**Fig. 2:**
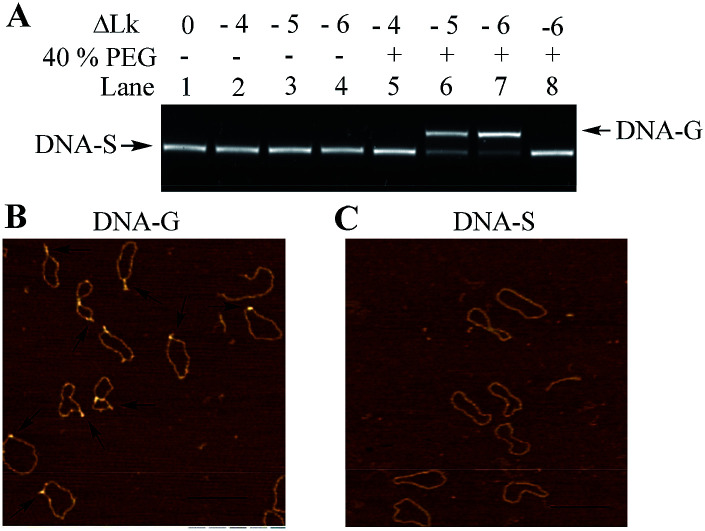
Examination of G-quadruplex formation in DNA topoisomers. (A) Electrophoretic analysis of DNA products. Lane 1: DNA-S alone; negative supercoiled DNA topoisomers (ΔLk = −4 to −6) were incubated in the solution containing 150 mM KCl and 4 mM NaCl in the absence (Lane 2–4) or presence (Lane 5–7) of 40% (w/v) PEG 200; Lane 8: DNA-C (no G-rich sequence containing) was treated with the same procedure as the one in Lane 7. (B) and (C) Structural confirmation of DNA-G and DNA-S using AFM. The DNA samples used for those AFM examinations were purified from the bands in Lane 7, scale bar 200 nm.

The negatively supercoiled DNA circles were subsequently incubated under physiological concentrations of potassium ions^25,26^ (150 mM KCl and 4 mM NaCl at pH 7.5) and molecular crowding condition created by PEG^21^ at 37 °C for 2 hours. At this stage, DNA supercoils and non-B structures may coexist in DNA circles, which make it difficult to compare the structural difference between the substrate DNA and products. It has been confirmed that G-quadruplex is a thermodynamically stable structural entity and once it formed G-quadruplex keeps intact even the additional negative supercoiling is removed.^14^ The rest of supercoils in DNA products were accordingly relaxed next using nicking endonucleases and further sealed the breaks using DNA ligase. As shown in Fig. 2A, the band in Lane 1 indicates the relaxed substrate DNA (DNA-S). No mobility shift difference can be observed in Lane 2 to 4, which suggests that no thermo-stable intramolecular secondary structure formed and negatively supercoiling alone cannot promote G-quadruplex formation in a potassium ion containing buffer. The result is consistent with Sekibo and Fox's reports.^15^ However, the slower moving bands (DNA-G) can be found in Lane 6 to 7 when molecular crowding condition created by PEG was applied. It has been confirmed that formation of G-quadruplex in DNA negative supercoil can alter the compactness of the DNA structure. The appearance of slower moving bands suggests that G-quadruplex structures were produced. In addition, the increasing of the amount of new bands in Lane 6–7 indicates that formation of G-quadruplex underwent a sharp transition over the superhelical density of −0.05 and −0.06. The ratio of the new bands in total DNA samples was also calculated by measuring the bands density in Lane 7. It showed that up to 73% of DNA-S was transformed into DNA-G when six supercoils were induced. In order to further confirm that the observed new bands are associated by the formation of G-quadruplex, a new DNA circle (DNA-C) was designed and synthesized. DNA-C is identical to DNA-S except that the potential G-quadruplex forming sequence was disrupted and replaced with a different 49 bp random sequence. The same procedures as the ones shown in Lane 7 in Fig. 2A were subsequently carried out in our studies except that DNA-S was replaced with DNA-C. As shown in Lane 8, no additional band can be observed, which indicates that slower moving bands in Lane 6–7 is indeed caused by G-quadruplex formation. AFM has been widely used to detect the subtle changes of the secondary and tertiary structures of DNA.^27^ The upper and lower bands were accordingly purified from the gel and examined by AFM. As shown in Fig. 2B, some raised structures (*e.g.*, spurs and blobs) can be observed in the DNA sample purified from the upper band. On the other hand, the circular DNA isolated from the lower band exhibited smooth backbone in the AFM images (Fig. 2C). The observations shown above indicate that G-quadruplex structure indeed exists in DNA-G. In addition, the stability of DNA-G in a dilute solution was also examined. As shown in Fig. S3 and S4,† no mobility difference can be found when the G-quadruplex-containing DNA was incubated in the solution with or without 40% PEG.

**Fig. 3:**
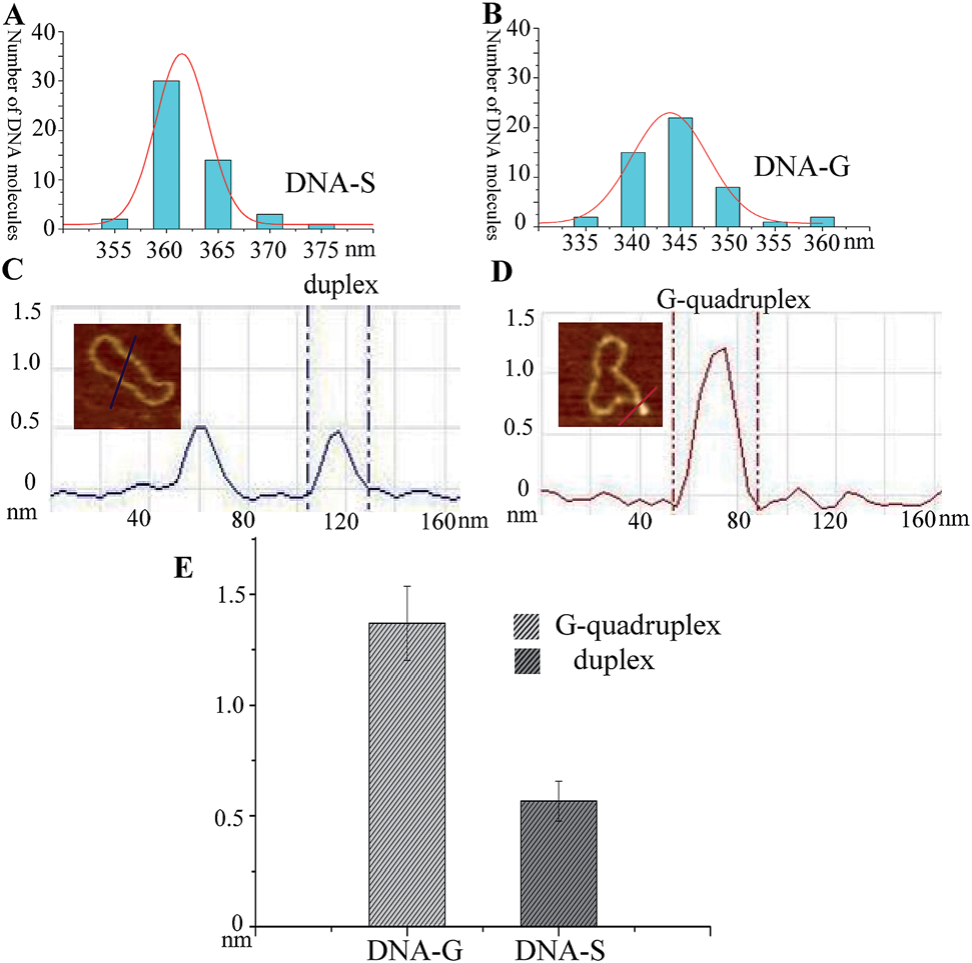
Comparison of the length and height of DNA-S and DNA-G. (A) and (B) Frequency distributions of the lengths (nm) of DNA-S and DNA-G in their AFM images. The curves indicate the fitted Gaussian functions. (C) Section analyses of two duplex DNA strands in DNA-S. (D) Section analyses of a G-quadruplex in DNA-G. (E) Histogram of the difference between the height of G-quadruplex in DNA-G and duplex in DNA-S.

It has been well studied that formation of intrastrand secondary structure causes the decrease of contour lengths of DNA circles.^28^ The circumferences of DNA molecules in Fig. 2B and C were accordingly measured. A series of very short lines along the DNA contour were set and the length of DNA circle was obtained by summating the length of each short line.^29^ The mean length was given through measuring fifty DNA molecules and each measurement was repeated three times. As shown in Table 1, the mean length of DNA-C (+SE) is 364.2 ± 4.2 nm, which gives the nm-to-bp conversion factor to be 0.35 (nm/bp) that is consistent with to the previous report of duplex DNA molecules measurement under dry AFM imaging.^28^ Since no non-B forming sequence is set in DNA-C, the result implies that the B-form is the predominant conformation and no apparent intrastrand secondary structure is formed in DNA-C. However, the mean length of DNA-G is 344.3 ± 3.0 nm, which is 18.2 nm (equivalent to ∼52 bp) shorter than that of the substrate DNA-S (362.5 ± 3.8 nm). The result suggested that the duplex DNA segment of 52 bp in DNA-S molecules is separated and the number is close to the G-rich sequence (49 nt). In addition, frequency distributions of circular DNA circumference are also given in Fig. 3A and B, which clearly shows the decrease of the length of DNA-S after it was transformed into DNA-G caused by the negative supercoils introduction under molecularly crowded condition.

**Table 1:** Quantization of length and height of DNA-G, DNA-S and DNA-C within fifty DNA molecules

Name	Spurs or blobs (>1 nm, %)	Contour length	Height of duplex	Height of G-quadruplex	N
DNA-G	83	344.3 ± 3.0 nm	0.53 ± 0.07 nm	1.37 ± 0.17 nm	50
DNA-S	2	362.5 ± 3.8 nm	0.56 ± 0.11 nm	N.A.	50
DNA-C	0	364.2 ± 4.2 nm	0.57 ± 0.09 nm	N.A.	50

Since the protrusions such as spurs and blobs were frequently observed alone the backbone of DNA-G, section analysis was performed on the raised spur and duplex. As shown in Fig. 3C and D, the height of spur is ∼2 times greater than that of duplex, which implies that the raised structure in DNA-G is G-quadruplex that is made up of four DNA strands. In addition, the height values of right raised structures (G-quadruplex) and duplex were also measured. In the case of spurs, the observed shapes were significantly different from anything seen on pure duplex DNA. As a result, all of these structures were included in the dataset. However, small raised structures (blobs) were occasionally seen on pure duplex DNA because of the variations in the imaging surface and/or kinks in the circular DNA. To distinguish the newly formed G-quadruplex structures from the features occasionally found on the pure duplex DNA, a criterion was set according to previous the studies.^29^ Generally, the normal height and the peak height were determined for 50 duplex DNA molecules (DNA-C). The mean of normal height (+SE) was 0.57 + 0.09 nm, and the mean of peak height was 0.61 + 0.07 nm, with a highest absolute value of 0.81 nm. Consequently, any blob <0.9 nm in height was excluded from the dataset and any blob >1 nm was included. The height measurements were taken across the base of each spur and the middle of each blob. As shown in Fig. 3E and Table 1, the mean of height (+SE) of DNA-G is 1.37 + 0.17 nm and 83% AFM images of DNA-G contain G-quadruplex structures. However, the raised structures (>1 nm) can be detected in only 2% of DNA-S.

**Fig. 4:**
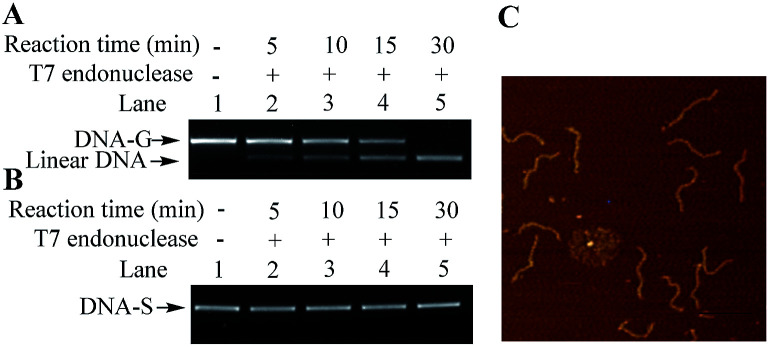
Endonuclease digestion assays on DNA-G and DNA-S. (A) Lane 1: DNA-G alone; Lanes 2 to 5: T7 Endonuclease I-catalyzed reaction products obtained by incubating DNA-G with T7 Endonuclease I at 37 °C for 5 min (Lane 2), 10 min (Lane 3), 15 min (Lane 4), and 30 min (Lane 5). (B) The same procedures were performed except that DNA-G was replaced with DNA-S. (C) AFM images of linear DNA obtained from the reactions of DNA-G with T7 Endonuclease I. The DNA sample used for the AFM examination was purified from the lower band in Lane 5 in (A), scale bar 200 nm.

Besides electrophoresis analysis and AFM measurement, the presence of G-quadruplex in DNA-G was also confirmed by endonuclease digestion assay. T7 Endonuclease I is a type of enzyme that has a special ability to cleave the non-perfectly matched DNA. Since the formation of G-quadruplex causes the non-perfect matched regions at the junction of duplex and G-quadruplex, circular DNA-G could be in theory cleaved by T7 endonuclease to produce the linear DNA.^14^ As shown in Fig. 4A, a new band with a faster rate of mobility shift was observed after DNA-G was treated with T7 endonuclease. On the other hand, no degradation product was found when DNA-S was incubated in the same reaction condition (Fig. 4B). Further AFM examination on the DNA isolated from the lower band in Lane 5 in Fig. 4C unveiled that the DNA product is linear, which indicated that DNA-G was cut by T7 Endonuclease I due to the presence of non-B structures. The results signify that double helix structure along the backbone of DNA-S keep integrate while non-B structures exist in the molecular structure of DNA-G.

## Conclusions

In summary, G-rich containing DNA topoisomers with different negative superhelical density were constructed and the possibility of G-quadruplex was investigated. Our results signify that G-quadruplex formation is driven by the combined actions of DNA negative supercoiling and molecular crowding condition. It has been reported that DNA purified from bacterial cells keep their superhelical density around −0.06 and one supercoil occurs in every 90 to 180 base pairs (*σ* ≈ −0.058 to −0.12) in nuclear DNA isolated from human dells.^11,30^ Our results provide clear structural evidence of G-quadruplex formation and suggest that G-quadruplex might generate from guanine-rich duplex DNA at physiological conditions to comply with the subsequent cellular functions.

## Conflicts of interest

There are no conflicts to declare.

## Supplementary Material

RA-009-C9RA06370F-s001
