# Supplementary material for: Formation of G-quadruplex structure in supercoiled DNA under molecularly crowded conditions
Source: RSC Adv. 2019 Aug 21;9(45):26248–51. doi: 10.1039/c9ra06370f (PMC9070399; doi:10.1039/c9ra06370f)
Supplement: RA-009-C9RA06370F-s001 [file RA-009-C9RA06370F-s001.pdf]

Electronic Supplementary Material (ESI) for RSC Advances.
This journal is © The Royal Society of Chemistry 2019

Supplementary Information

Formation of G-quadruplex structures in supercoiled DNA under molecularly crowded condition

Dawei Li, *[a] Peiwen Peng,[a] Zhaoqi Yang[c] and Bei Lv*[b]

EXTENDED EXPERIMENTAL PROCEDURES

Reagents: *taq* DNA polymerase, T4 DNA ligase, Sac I, Nt.BsmAI, T7 endonuclease were purchased from New England Biolabs (Ipswich, MA). Plasmid X2547 for PCR were provided by Generay Biotech (Shanghai, China). PNAs were purchased from Bio-Synthesis (Lewisville, TX). Primers were provided by Sangon Biotech (Shanghai, China) with HPLC purification. All the buffer and solution are prepared by the biological purity water.

Polymerase chain reaction: Polymerase chain reaction was carried out following standard procedures with *taq* DNA Polymerase. Primers for liner DNA 1: ssODN-1 (5'-3': CCGAGCTCCCTGGTCCCCACACTCCCAACCTACCCCAGCTCCCCCAGAGCTGCCCAG TACATGTGCTGAGGATCGAG) and ssODN-2 (5'-3': CCGAGCTCTCGTTTGGTATGGCTTCATT).

Reactions of SacI with Linear DNA: A solution containing 10 mM Bis-Tris-Propane-HCl, 10 mM $MgCl_2$, 1 mM Dithiothreitol, linear DNA (500 ng) and 10 U SacI was incubated at 37 ℃ for 1 hr.

Preparations of DNA-S: A 50 μl solution containing 50 mM Tris-HCl, 10 mM $MgCl_2$, 1 mM ATP, 10 mM dithiothreitol, 500 ng linear DNA with cohesive ends and 20 U T4 DNA ligase was incubated at 16 ℃ for 8 hrs.

Reactions of Nt.BsmAI with DNA-S: A solution that contained 20 mM Tris-acetate, 50 mM potassium acetate, 10 mM magnesium acetate, 1 mM dithiothreitol, 200 ng of DNA-S and 10 U Nt.BsmAI at 37 ℃ for 1 hour.

Reactions of T7 endonuclease I with DNA circles: A 50 μl solution containing 50 mM NaCl, 10 mM Tris-HCl (pH = 7.9), 10 mM $MgCl_2$, 1 mM DTT, 500 ng circular DNA and 0.5 U T7

endonuclease I was incubated at 37 ℃ for 5 to 30 min. The obtained products were further analyzed using agarose electrophoresis (1.0 %) in the absence of ethidium bromide. The gel was photographed and the DNA bands were measured and quantified using Gel Documentation System (BioRad ChemiDocXRS, US).

PNA invasion: In the binding reactions of PNA 1 to target sites, the PNA concentration was kept at a large excess over the DNA concentration, and the binding was performed at 37 ℃ for 6 hr in 10 mM Sodium-Phosphate Buffer (PH = 6.9)[1].

AFM studies: AFM examination was conducted following reported procedures and a specially prepared mica surface was selected as the substrates for DNA binding.[2, 3] Generally, the micas used in the our studies were modified on their surfaces with (3-aminopropyl)triethoxysilane (APS). Sample preparation procedures are described as follows: 5 μl to 10 μl of solutions containing 20 mM Tris-HCl (pH = 7) and 0.1 to 0.01 μg/ml DNA were dropped into the middles of the newly prepared APS-mica plates (1 x 1 cm^2), which were further kept at room temperature for 5 minutes. 10 ml of distilled water were then used to rinse the APS-mica plates that has been bound by DNA molecules for 3 times in order to remove the salt and buffer. Before scanning, the samples were placed in vacuum desiccator for 30 min. AFM images of DNA molecules on the APS-mica plates were obtained in Tapping Mode™ on a Dimension Edge™ AFM (Bruker, Santa Barbara, CA) in connection with a Nanoscope VIII™ controller. Aluminum reflective coating cantilevers with nominal spring constants between 1 and 5 N/m were selected. Scan frequency was 1.9 Hz per line and the modulation amplitude was in a nanometer range. All DNA sample determinations were carried out in air at room temperature. The lengths (in nm) of DNA were obtained by detecting the circumference alone the backbone of circular DNA, which were measured by drawing a series of very short lines along the DNA contour and summating the

lengths. Height and width measurements of DNA strands were performed manually using the software provided by the Nanoscope instrument. Fifty molecules were analyzed for each sample. Mean heights were obtained by fitting to a Gaussian curve.

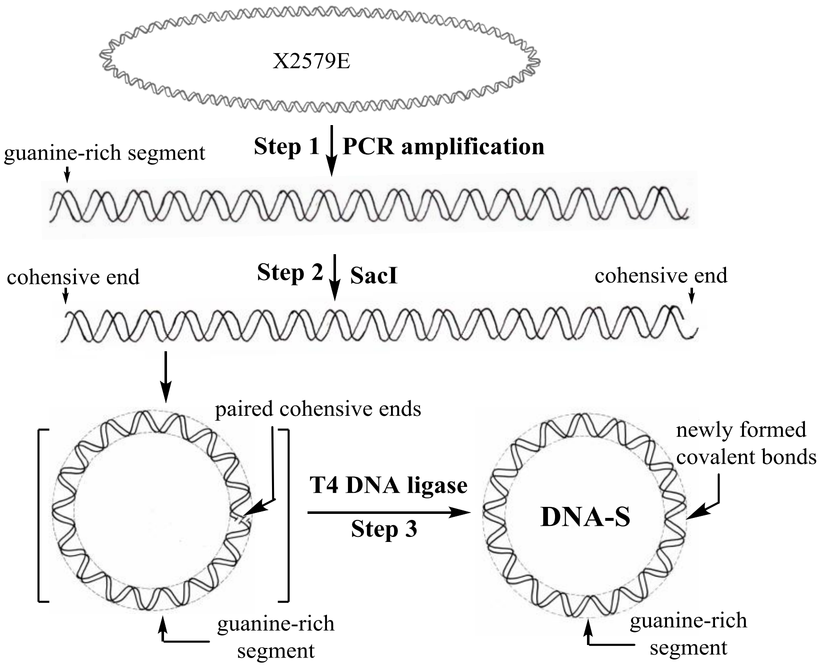

Fig. S1 Diagrammatic illustration of synthesis of DNA-S. Step 1: Linear DNA with a G-rich segment was obtained by PCR amplification; Step 2: Linear DNA with two cohesive ends was obtained by SacI digestion; Step 3: DNA-S was produced by the reaction catalyzed by T4 DNA ligase.

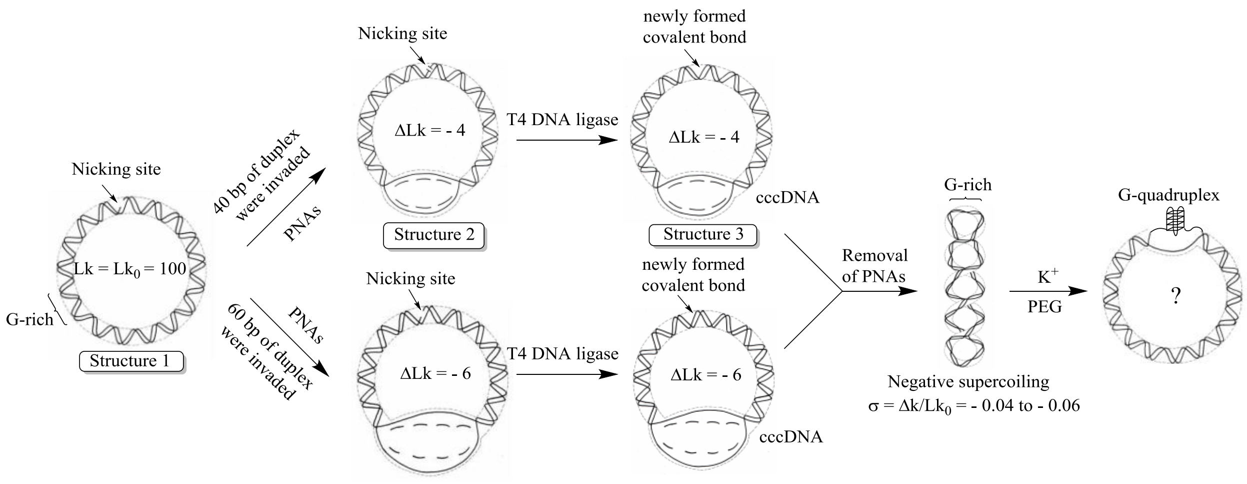

Fig. S2 Diagrammatic illustration of engineering of DNA supercoils on the basis of PNA invasion and G-quadruplex formation. If a circular DNA possesses 1040 base pairs in length, for example, the Lk_0 value of this DNA (Structure 1) should be 100 (1040/10.4 = 100) because the DNA circle with nicking site is in the relaxed conformation. When a designed PNA-1 invades the target nicking site-containing circular DNA that contains the complementary segments (40 bp in length) to the PNA-1, the linking number of the target DNA (Structure 2) will be reduced to 96 from 100 and the linking number difference (ΔLk) value should be - 4 ($Lk - Lk_0$ = 96 - 100 = - 4). This happens because the helical turns in PNA binding sites of circular DNA are interrupted and incapable of maintaining the regular double helicity any longer. After sealing the nicking site with T4 DNA ligase and removal of the 40 base PNAs, a covalently closed circular DNA (cccDNA, Structure 3) can be obtained and the ΔLk value in this circular DNA is still -4 and no further linking number difference can be changed. Therefore, a supercoiled DNA with superhelical density of - 0.04 is produced ($\sigma = \Delta Lk/Lk_0$ = - 0.04). If more PNA binding sites were designed, higher level of negative supercoiling can be generated in substrate DNA.

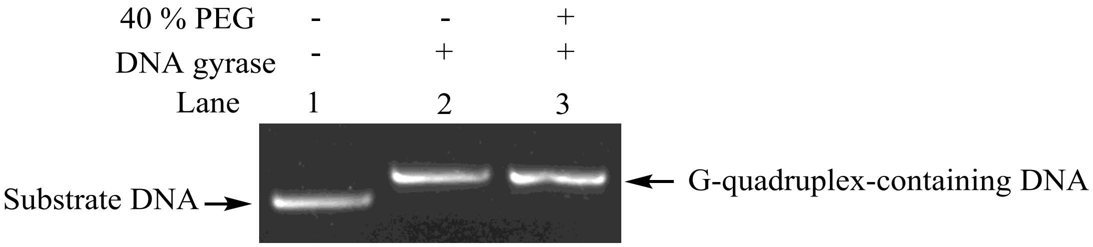

Fig. S3 Electrophoretic analysis of G-quadruplex-containing DNA obtained by DNA gyrase treatment in the presence or absence of 40% PEG. Lane 1: Substrate DNA (G-rich sequence containing); Lane 2: G-quadruplex-containing DNA obtained by DNA gyrase treatment with substrate DNA under physiological concentrations of potassium ions (150 mM KCl and 4 mM NaCl at pH 7.5); Lane 3: DNA product in Lane 2 was incubated under molecular crowding condition created by 40% PEG at 37 ℃ for 2 hours.

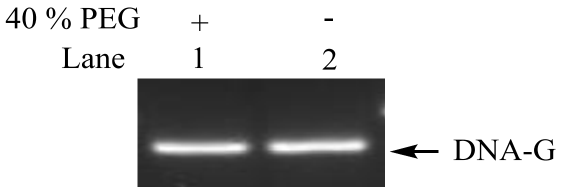

Fig. S4 Electrophoretic analysis of incubation of DNA-G in the presence or absence of 40% PEG. Lane 1: DNA-G incubated in a solution with 40 % PEG (150 mM KCl, 4 mM NaCl and 40 % (w/v) PEG 200 at pH 7.5); Lane 2: DNA-G incubated in a solution with 40 % PEG (150 mM KCl and 4 mM NaCl at pH 7.5).

Tab. S1 Nucleotide sequences of bis-PNAs.

bis-PNA	sequence
PNA-1	H-$(Lys)_2$TJTJJTJJTT-$(eg1)_3$-TTCCTCCTCT-Lys-NH_2
PNA-2	H-$(Lys)_2$TTJJTJTJTT-$(eg1)_3$-TTCTCTCCTT-Lys-NH_2
PNA-3	H-$(Lys)_2$TTJTTJTTTT-$(eg1)_3$-TTTTCTTCTT-Lys-NH_2

Note: bis-PNAs are written from the N terminus to the C terminus using normal peptide conventions: H is a free amino group; NH2 is a terminal carboxamide; Lys is the lysine residue; J denotes pseudoisocytosine and eg1 denotes the linker unit, 8-amino-3,6-dioxaoctanoic acid.

Tab. S2 Nucleotide sequence of DNA-S. Sequence only shows one strand from 5' to 3'. Gray shadow indicates a 49 bp G/C-rich fragment of the murine Sγ3 switch region. The sequences in yellow shadow are the binding sites for PNA-1. The sequences in red and blue shadow are the binding sites for PNA-2 and PNA-3 respectively.

```
<5'GAGCTCCCTGGTCCCCACACTCCCAACCTACCCCAGCTCCCCCAGAGCTGCCCAGTACATGTGCTGAGGAT
CGAGTTAATTACTGCGCCTTGTAGAAACGCAAAGGCCATCCGTCAGGATGGCCTTCTGCTTAGTTTGATGCCTG
GCAGTTTATTTGCTTCACACCCGGCGATTTGTCCTACTCAGGAGAGCGTTCACCGACAAACAACAGATAAAACG
AAAGGCCCAGTCTACCGACTGAGCCTTTCGTTTATTTGATGCCTGGCAGTTCCCTACTCTCGCGTTAACGCTAG
CATGGATGTTTTCCCAGTCACGACGTTGTAAAACGACGGCCAGTCTTAAGCTCGGGCAAATAATGATTTTATTG
ACTGATAGTGACCTGTTCGTTGCAACAAATTGATGAGCAATGCTTTAAGGAGGAGACGCAAGGAGGAGATTTAT
AATGCCAACTTTGTACAAAAAAGCAGGCTTGAAGGAATTCGGCAAGTCTTCCCACGCGGGAAGCTTGTACGTTG
CCAAGGAGGAGACGTAAGGAGGAGACGACCGGTCGACCTCGAGAAGCTTGCGCGGCCAACCTAGGTATCTAGAA
CCGGTCTCGAGCCATAACTTCGTATAGCATACATTATACGAAGTTATAAGAGAGGAGGCGGAAAAGAAGAAGCT
GTCAAACATGAGAATTCTTGTTATAGGTTAATGTCATGATAATAATGGTTTCTTAGACGTCAGGTGGCACTTTT
CGGGGAAATGTGCGAACCCCTATTTTCTAAATACATTCAAATATGTATCCGCTCATGAGACAATAACCCTGATA
AATGCTTCAATAATATTGAAAAAGGAAGAGTATGAGTATTCAACATTTCCGTGTCGCCCTTATTCCCTTTTGCG
GCAAGAAACGCTGGTGAAAGTAAAAGATGCTGAAGATCAGTTGGGTGCACGAGTGGGTTACATCGAACTGGATC
TCAACAGCGGTAAGTTAAGCTTTTTGCACAACATGGGGGATCATGTAACTCGCCTTGATCGAATGAAGCCATAC
CAAACGA 3'>
```

Note: (1) DNA-S is a circular DNA; and

(2) < and > stand for the termini that are covalently connected.

References for Supplementary Information:

1. D. Li, Z. Yang, Y. Long, G. Zhao, B. Lv, S. Hiew, M. T. Ng, J. Guo, H. Tan, H. Zhang, W. Yuan, H. Su and T. Li, *Chemical communications*, 2011, **47**, 10695-10697.
2. A. L. Pyne and B. W. Hoogenboom, *Methods Mol Biol*, 2016, **1431**, 47-60.
3. D. Li, B. Lv, Q. Wang, Y. Liu and Z. G. Qiang, *Bioorg Med Chem Lett*, 2017, **27**, 4086-4090.
